# The development of neural responses to emotional faces: A review of evidence from event-related potentials during early and middle childhood

**DOI:** 10.1016/j.dcn.2021.100992

**Published:** 2021-07-21

**Authors:** Felicity J. Bigelow, Gillian M. Clark, Jarrad A.G. Lum, Peter G. Enticott

**Affiliations:** Cognitive Neuroscience Unit, School of Psychology, Deakin University, Geelong, Australia

**Keywords:** Facial emotion processing, Early childhood, Middle childhood, Event-related potentials, Systematic review, Meta-regression

## Abstract

Facial emotion processing (FEP) develops throughout childhood and provides crucial social information necessary for the interpretation and prediction of others’ behaviour. This systematic review, which includes a meta-regression component, sought to explore the development of FEP event-related potentials (P100, N170, and late positive potential [LPP]) in children aged three to twelve years. Thirty-four studies, representing data from 1511 children, were included in the review. The combination of meta-regression and systematic review suggest that P100 amplitude decreases with increasing age in response to emotional facial stimuli. P100 latency may show a gradual decrease around the age of ten. In terms of the N170, it is suggested that amplitude follows a non-linear trend with age, and latency may decrease in early childhood before plateauing during middle childhood. Of note, review of the literature indicates that substantial methodological differences and high levels of heterogeneity exist. We suggest future research considers these results within the context of emotion-specific development, whilst also acknowledging how this may relate to individual social functioning skills across early-to-middle childhood.

## Highlights

• We examine the development of facial emotion processing (FEP) event-related potentials (ERPs) in early-to-middle childhood.
• We provide a systematic review with a meta-regression component across 34 studies.
• FEP ERPs continue to develop into middle childhood.
• Results suggest the development of FEP may differ to previously reported findings of processing neutral faces..
• Substantial methodological differences accentuate need for additional research.

## 1 Introduction

From very early childhood, humans possess a distinctive and specialised ability to identify and differentiate emotional facial expressions (Posamentier and Adbi, 2003). This ability has a number of adaptive benefits, but broadly speaking it can provide context and understanding to otherwise ambiguous social environments. Facial emotion processing (FEP) is defined as the neural and cognitive functions involved in the recognition of an emotional facial expression. This skill encapsulates the processes involved in detecting and attributing affective meaning to facial stimuli and is often considered an *affective* theory of mind (Sebastian et al., 2012).

Social skills that rely on FEP undergo considerable development across early-to-middle childhood. This period includes children within the ages of three to twelve years (Ostrov and Godleski, 2010). Early childhood, typically represented as existing until the age of eight years, is a time of rapid development across foundational skills of language, gross and fine motor movement, and cognition. Whilst there is no general consensus for the definition of middle childhood, it is important to differentiate between developmental periods in childhood. Therefore, for the purposes of this review, middle childhood is defined as ages 9–12 years, and is synonymous with the integration of existing developed skills within a social context (Lecce et al., 2019). An emphasis on meaningful and increasingly complex and interpersonal communications, coupled with a heightened and rapidly expanding independence, renders early-to-middle childhood a critical social developmental epoch. Underlying neural networks implicated in the processing of affective stimuli gradually develop an interactive specialisation throughout childhood. Whilst children begin to display relatively similar neural patterns to that of adults by the age of four years, findings indicate that significant development occurs throughout childhood (Grossman and Johnson, 2007; Johnson et al., 2005). Compared to infant and adolescent stages, however, FEP across the important developmental period of early-to-middle childhood has received relatively little focus within the literature.

### 1.1 Event-related potentials during FEP

The neural processes that underpin the emotional processing of faces can be studied using event-related potentials (ERPs; Bentin et al., 1996). Previous studies have demonstrated that certain ERP components exhibit heightened neural reactions to human faces, and seem to be modulated by emotional expression (Dawson et al., 2002). By analyzing face-sensitive ERPs, the speed with which these processes occur (latency) and the degree of activity (amplitude) during FEP can provide developmental indicators of social cognitive maturation. Alongside maturation, one would speculate a decrease in both latency and amplitude, possibly reflective of neural specialisation, myelination, and enhanced neural efficiency.

**Fig. 1:**
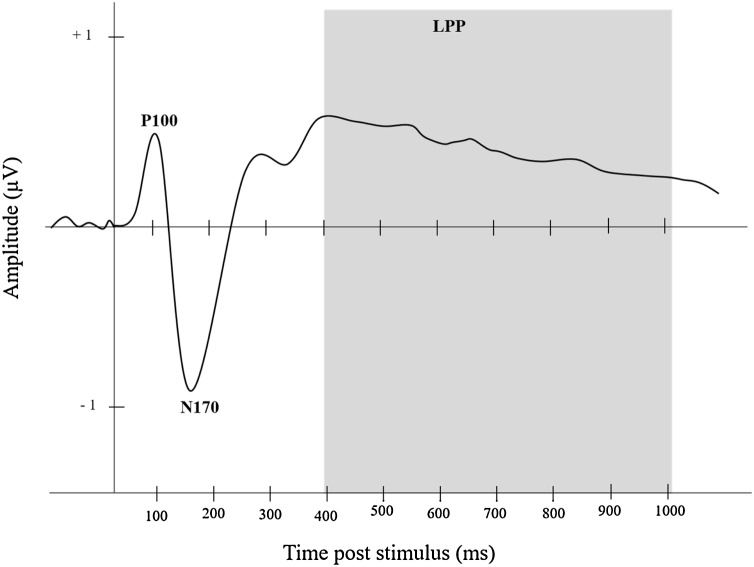
Example of an Averaged ERP Waveform Labelled with Face-Specific Components P100 and N170 Labelled, and LPP Highlighted in Grey.

There are three ERP components that seem critical to understanding the neurophysiology of FEP (shown in Fig. 1). The P100 is a positive deflection, maximal at occipital regions, that occurs roughly 100 ms post stimulus onset (Herrmann et al., 2005). In children, the P100 is often recorded between 90−150 ms (Luyster et al., 2017; Young et al., 2017). The P100 reflects general visual processing activity, although there is some evidence that it may additionally relate to the initial processing of facial configuration (de Haan et al., 2003). Indeed, some research has found the P100 to display distinctive activity to faces rather than objects in children as young as four years old (Kuefner et al., 2010).

The N170 is a negative peak, maximal at posterior regions, that occurs roughly 170 ms after stimulus onset (Bentin et al., 1996). In children, the N170 is often recorded between time windows extending to 150−300 ms (Batty and Taylor, 2006). This component typically reaches a maximum amplitude at lateral occipito-temporal electrode sites, and is most prominent in the right hemisphere (Eimer and Holmes, 2007). The N170 possesses a heightened amplitude for facial stimuli when compared to inanimate objects, and is hypothesised to be reflective of higher-level processes, including, but not limited to, the perception of a face (Posamentier and Adbi, 2003). The N170 is modulated by facial emotion expression, with larger amplitudes recorded for negative emotions (Batty and Taylor, 2006; Hinojosa et al., 2015). Development of the N170 (in terms of changes in latency and amplitude) continues throughout childhood. Nevertheless, some research has suggested that the beginnings of this face-sensitive ERP are apparent from the age of four years (Kuefner et al., 2010; Taylor et al., 2001).

The Late Positive Potential (LPP) is a slow positive wave at centro-parietal regions that begins roughly 400 ms post-stimulus onset (MacNamara et al., 2016). The LPP can endure for several seconds and thus is often categorized into early, middle, and late windows (Hajcak et al., 2009). The LPP is largest at occipital sites in early childhood, before moving towards parietal sites in middle childhood. The LPP has repeatedly been shown to exhibit heightened activity for emotional stimuli when compared to neutral stimuli in children as young as six years of age (Bunford et al., 2017; Kujawa et al., 2013; Wessing et al., 2011). Evidence suggests that the LPP is associated with higher-order processes reflective of selective attention towards affective stimuli, including emotional faces (Grunewald et al., 2018; Keil et al., 2018).

### 1.2 FEP ERPs during child development

The P100, N170, and LPP differ in amplitude and latency from preschool years to adolescence, indicating that neural development underpins these components (Chronaki et al., 2018). Development of the P100 may reflect a heightened ability to identify, process and attend to visuo-spatial stimuli, as illustrated through reductions in amplitude and latency (Batty and Taylor, 2006). It has been suggested that the N290 and P400 ERP components observed in infants are precursors for the later emerging face sensitive N170 (Leppänen et al., 2007). Therefore, the development of the N170 in childhood may initially display a bifid shape, before the merging of two neural regions, thought to later establish the singular face specific ERP (Webb et al., 2011). Indeed, it is possible that the development of the N170 may suggest a heightened interactive specialisation between neural regions implicated within FEP (Taylor et al., 2004). In other words, the development of a stronger N170 amplitude and a reduced N170 latency may indicate heightened expertise in the processing of facial emotions (Hileman et al., 2011). Previous literature has shown that LPP amplitude tends to decrease alongside development (MacNamara et al., 2016). LPP amplitude development during childhood may indicate reductions in the allocation of attentional resources required to process facial emotional stimuli (Keil et al., 2018). Furthermore, LPP development may reflect greater expertise in cognitive reappraisal skills (Dennis and Hajcak, 2009).

While numerous studies have investigated ERPs as a response to facial stimuli in childhood, few have examined the influence of age on these ERPs. In an early review examining the neurophysiological response to faces, Taylor et al. (2004) synthesised findings from four ERP studies exploring (primarily neutral) facial stimuli. It was suggested that the latency of the P100 and N170, and the amplitude of the P100, decreased with age across childhood, whilst N170 amplitude appeared to follow a non-linear pattern. During childhood, N170 amplitude seemed to initially decrease (become less negative) from the age of four years until roughly 10−13 years of age, before then increasing (i.e., becoming more negative) towards similar activity observed like that in adults. Data from children aged four to seven years old, however, came from only one study. Furthermore, the four studies included in the review used different face-processing tasks, which was also found to influence the amplitude and latency of the ERPs. For example, while Taylor et al.’s (2004) review focused only on neutral face stimuli, data for one study were taken from a task that displayed both neutral and emotional faces. It was highlighted that when the task contained emotional faces, ERP latencies were shorter, with larger amplitudes. This suggests that the development of FEP ERPs may differ across emotion categories. Overall, the extent to which Taylor et al.’s (2004) findings reflect the broader literature, and particularly how they relate to expressive faces, remains unclear.

More recent studies have further investigated the development of FEP ERPs across ‘basic’ emotions of happiness, sadness, disgust, fear, anger, and surprise. This is important, as human faces are often expressive, as opposed to entirely neutral, and can provide an individual with an adaptive advantage to predict potentially threatening situations (Eimer et al., 2003). The development of these FEP ERPs during early-to-middle childhood may pose broader implications. Previous research has illustrated correlations between FEP ERPs during early-to-middle childhood and individual emotional skills (Hileman et al., 2011). For example, Hoyniak et al. (2019) reported that children aged three to five with higher levels of unemotional traits displayed reduced N170 amplitudes when viewing fearful facial stimuli. Additionally, Chronaki et al. (2018) found that children aged five to 11 years with higher levels of trait anxiety and depression, recorded larger LPP amplitudes to angry facial stimuli. This indicates that FEP ERPs may provide insight into wider social emotional functioning and development.

Among the studies exploring FEP ERP development, some report an effect of age, with smaller amplitudes and latencies with increasing age (Batty et al., 2011; Batty and Taylor, 2006; Meaux et al., 2014), while others find no influence of age (Battaglia et al., 2007), or an inverse effect (Chronaki et al., 2018). A common limitation within these studies are the small sample sizes within each age group, which limits the ability to detect differences between ages. Furthermore, variables that differ across studies, such as the emotional expression and stimulus presentation, may influence the components, making cross-study comparisons difficult. Given the numerous methodological differences, a comprehensive and systematic review that summarises the development of FEP ERPs would be of significant benefit to developmental cognitive neuroscience.

The primary aim of this systematic review was to chart the developmental course of the P100, N170 and LPP response to emotional faces throughout early-to-middle childhood (3–12 years). This review used meta-regression to examine age related changes in the aforementioned ERP components. In this review, meta-regression allowed the synthesis of data from 21 studies, representing 804 participants. A limitation of meta-regression, however, is the use of the average age of a sample, which may mean subtle age effects are overlooked. Therefore, this review additionally summarised all studies that reported analyses specifically investigating the influence of age on FEP ERPs. While the individual studies may have small sample sizes, methodology within a study is constant, and age effects may therefore be clearer. It was hypothesised that the P100 latency and amplitude would decrease with age. It was also hypothesised that the N170 latency would decrease with age, whilst the N170 amplitude would become stronger (i.e., more negative) with age. Finally, it was hypothesised that the LPP amplitude would decrease with age.

## 2 Methods

### 2.1 Search strategy

A systematic literature search was undertaken to identify relevant literature. The electronic databases that were searched were Medline Complete, Psychological Information Database (PsychINFO), PsycExtra, Scopus, Web of Science, Excerpta Medical Database (EMBASE), and Informit. The search was initially performed on the 28th of February 2019, with a subsequent search performed on the 20th of April 2020. Additionally, alerts across all databases were created to ensure the inclusion of any later eligible studies. Databases were searched using the following search syntax: (((EEG) OR (electroencephalogra*) OR (ERP*) OR (electrophysiolo*) OR (“event-related potential”) OR (“event related potential”) OR (“evoked potential”)) AND ((facial) OR (face*)) AND ((child*) OR (school*))).

### 2.2 Selection criteria

Articles were included for review if they met the following criteria. Participants in the study were required to be typically developing children aged between three and twelve years. Thus, the mean age of each study’s sample was required to be between three years and zero months to 12 years and 11 months. To avoid the inclusion of underpowered and/or unrepresentative studies, the minimum overall sample size to meet inclusion criteria was set at 10. Each study was required to have implemented a visual facial processing task using photographs of at least two ‘basic’ emotions (i.e., happiness, sadness, disgust, fear, anger, surprise, and/or neutral) from an empirically validated set of visual facial stimuli. Studies were required to have collected EEG data during performance of the FEP task, and to have extracted at least one of the aforementioned ERPs that were epoched to facial emotion stimulus onset. To ensure studies remained homogenous as possible, only those presenting a single face stimulus, from a front profile with direct gaze, with no additional text or images overlaid across the face, were included. Task presentation was required to consist exclusively of the facial stimuli with no additional auditory stimuli, and no changes in spatial frequency of the stimuli. Both implicit and explicit tasks were included for this review. For the purposes of this review, an implicit FEP task required either no response from the participant, or a response that was not emotion-specific, such as determining the gender of the face (Herba et al., 2006). For a task to be deemed explicit, an emotion-related response, such as naming the expression of the stimulus presented, was required. Finally, papers were required to be published in English, and within peer-reviewed, academic journals.

### 2.3 Screening process

**Fig. 2:**
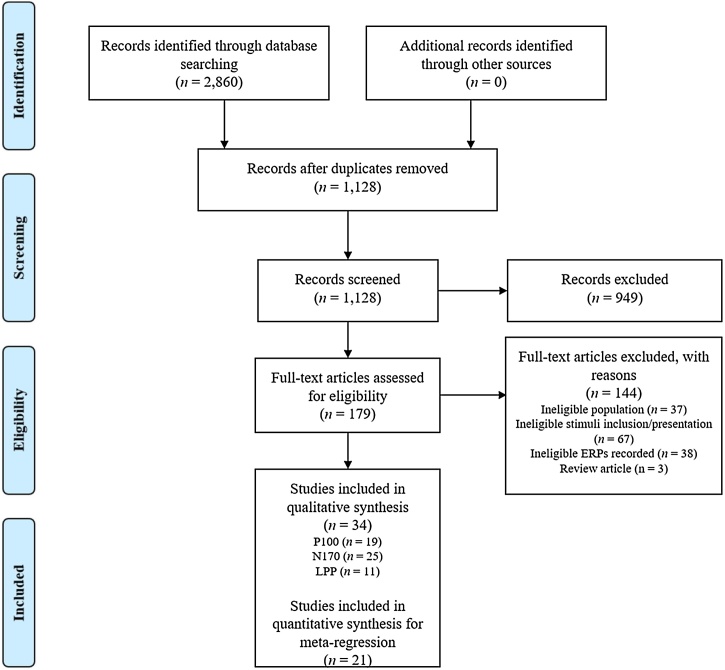
Prisma Flowchart outlining Screening Process.

The initial search yielded 2860 papers. Following the removal of duplicates, 1128 titles and abstracts were screened by author FB in accordance with the aforementioned study criteria. After the initial screening, 179 full-text articles were assessed for eligibility. A second author (GC) screened 10 % of articles. There was 100 % agreement on the eligibility of these studies. Fig. 2 summarises articles excluded following application of each criterion according to PRISMA guidelines (Moher et al., 2010). Overall, a total of 34 papers were found to be eligible for this review.

### 2.4 Data extraction

Three categories of data were extracted: 1) For the meta-regression, mean latency, peak amplitude, and the associated standard error were extracted or estimated (further details provided below) for each ERP component (P100, N170, and LPP), as well as the mean age of the sample, 2) methodological details, including the sample size, the stimulus set used and emotions presented, and the electrodes used to record the component of interest, and 3) for studies that directly analysed whether age influenced the ERP component latency and/or amplitude, the result of the analysis. The specific combination of facial expressions varied across studies; thus, an average of all expressions, including neutral stimuli, were used.

Several studies included multiple participant groups or conditions. For studies that included a clinical group, only data from typically developing control groups were extracted. This resulted in the exclusion of data from clinical groups of 19 of the 34 studies. Data were also excluded from the experimental condition in Burkhouse et al. (2019), and the post-intervention condition in Hum, Manassis, and Lewis (2013b). Another experimental manipulation is the use of implicit or explicit task designs. In comparison to implicit tasks, explicit tasks have been previously shown to produce heightened P100 amplitudes and shorter P100 and N170 latencies (Kliemann et al., 2013; Luckhardt et al., 2017; Saavedra et al., 2010). These differences may, in part, reflect the increased cognitive effort during explicit tasks (Wong et al., 2008). Considering the scope of this review, both explicit and implicit task studies were included in the systematic review. However, given previous research illustrating the influence that task design has upon FEP ERPs, only studies with implicit tasks were included in the meta-regression. One study by Wong et al. (2008) included both explicit and implicit tasks; only data from the implicit task were extracted for the meta-regression.

LPP amplitude was not included in the meta-regression analysis due to the small number of studies (*n* = 11) exploring this component. Additionally, data from those studies examining the LPP were not considered homogenous enough to analyse with meta-regression. Specifically, the time-windows used to define the LPP were different widths, with different onset and offset times across studies. Therefore, it was decided to include LPP amplitude solely in the systematic review section.

Several studies did not report the average ERP values and/or standard error, which are necessary for the meta-regression. As the omission of studies due to incomplete data may introduce bias, one of the following techniques were implemented to impute values. For some studies, values were provided by the author upon request, or were extracted from figures using Plot Digitizer Version 2.6.8. Seven studies did not report standard error, nor conduct analyses to allow estimation of standard error, for one or more ERP components. For each of these studies, the standard error value used was the median standard error of all of the included studies with comparable sample sizes (as recommended by Furukawa et al., 2006). To examine whether the meta-regression was sensitive to the use of median standard error values, additional analyses were undertaken. In these analyses, meta-regression was run using the minimum or maximum reported standard error of the comparable datasets for each of the studies with missing values. Results remained constant unless otherwise acknowledged. Refer to Supplementary material for descriptions of all meta-regression data extracted from each study.

### 2.5 Meta-regression

Before undertaking the meta-regression, we calculated the amount of heterogeneity between studies that is attributable to systematic influences (such as age or methodological differences), estimated with the *I*^2^ statistic. This statistic, reflected as a percentage, describes the proportion of variance across studies that is due to between-study error or ‘true heterogeneity,’ rather than sampling error (Borenstein et al., 2011). Larger *I*^2^ values indicate the presence of systematic influences on study findings. As a general guideline, values of 25 %, 50 % and 75 % can be considered as low, medium, and high levels of heterogeneity, respectively (Higgins et al., 2003). Four *I*^2^ values were calculated, for the latency and amplitude of both the P100 and N170, using Comprehensive Meta-Analysis Version 3 (Borenstein et al., 2013).

A random effects meta-regression was conducted to determine the proportion of between-study differences that are attributable to age. In the context of the current review, meta-regression was used to examine whether age predicted study level effect sizes. Four meta-regressions were calculated for the latency and amplitude of both the P100 and N170 using the METAREG macro (Wilson, 2005) available for the SPSS statistical package (IBM, Version 26). In this review, the average age of the dataset was entered as the predictor variable, and the average amplitude or latency for the dataset was the study-level effect size. Five studies reported data for several subgroups of different ages (Batty et al., 2011; Batty and Taylor, 2006; Bertoletti et al., 2012; Meaux et al., 2014; Miki et al., 2011). To increase the specificity of the amplitudes and latencies across the different ages, the subgroup mean ages and effect sizes, rather than the average for the whole sample, were entered into the meta-regression.

### 2.6 Risk of Bias

**Fig. 3:**
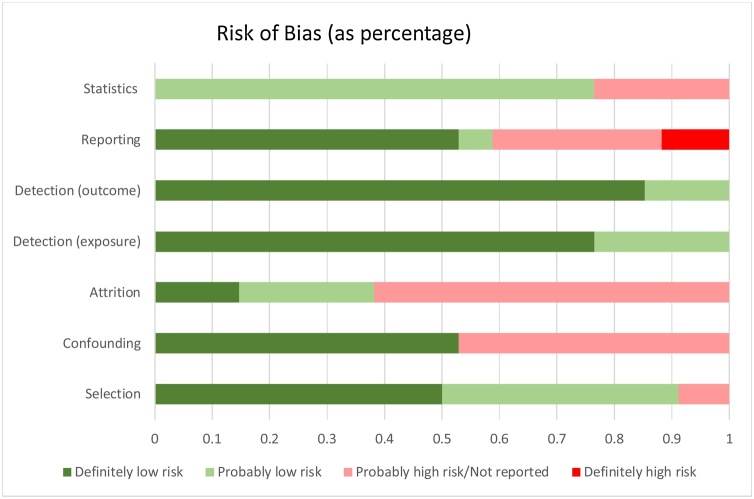
Risk of Bias Across Studies.

Studies included in this review were assessed for risk of bias using the NTP−OHAT Risk of Bias Assessment Tool (National Institute of Environmental Health Sciences., 2015). This tool assesses risk of bias across selection, confounding, attrition/exclusion, detection and selective reporting domains. Overall, results suggest that 30 of the 34 studies were assessed as having a probable risk of bias across at least one of the domains. As illustrated in Fig. 3, risk of bias was primarily associated with data analysis. As well as failing to report all assumptions associated with analyses, the majority of studies failed to report details on the exclusion or attrition of data. Furthermore, 4 of the 34 studies reported only significant results, whilst an additional 10 studies either did not report, or were assessed as having a probable high risk of bias in terms of the selective reporting of results. Refer to Supplementary material for risk of bias assessments for each individual study.

## 3 Results

### 3.1 Development of P100

Nineteen studies examined the P100 component. Table 1 summarises the methodology and results of these studies. Five studies investigated the relationship between P100 and age during early-to-middle childhood. Below is an overview of methodological aspects of these studies and relevant findings.

**Table 1:** Methodological Summaries of Studies within the Review that Reported P100 Amplitude and/or P100 Latency.

Author	n	Age range	Facial emotion processing task design elements					P100 Amplitude		P100 Latency	
			Stimulus set	Emotions	Presentation	Task	Electrode sites	Recorded [μV]	Age effects	Recorded [ms]	Age effects
Apicella et al. (2013)	12	6−13	NimStim	H, F, N	B&W; static; M, F; adults	Implicit	T5, T6, PO7, PO8, P9, P10	10.31	–	104.22	–
Batty et al. (2011)	30	4−15	From Batty and Taylor (2003)	H, A, Sa, Su, F, D, N	B&W; static; M, F; adults	Implicit	O1, O2				
Group 1	15	4−15						27.5	–	108.71	–
Group 2	15	5−15						22	P100 ↓ ***	103	P100 ↓***
Batty and Taylor (2006)	69	4−13	From Batty and Taylor (2003)	H, A, Sa, Su, F, D, N	B&W; static; M, F; adults	Implicit	O1, O2, PO9; PO10		P100 ↓***		P100 ↓ ***
Group 1	13	4−5						23.41	Groups: 1 > 3, 4, 5	117.86	Groups: 1 > 4***, 5***
Group 2	15	6−7						24.44	Groups: 2 > 4, 5	114.13	Groups: 2 > 4***, 5***
Group 3	13	8−9						19.69	Groups: 3 > 5	109.35	Groups: 3 > 4***, 5***
Group 4	13	10−11						16.14		103.54	
Group 5	15	12−13						14.74		108.13	
Chronaki et al. (2018)	58	5−11	POFA	H, A, N	Colour not stated; static; 2 F; adults	Explicit	Parietal & occipital sites	17.2	N. S	146.35	–
Curtis and Cicchetti (2011)	25	3−4	POFA	H, A, N	B&W; static; 1 of 3 F; adults	Implicit	O1, O2, Oz	18.13	–	134.45 Measured at Oz	–
D’Hondt et al. (2017)	15	3−5	NimStim	H, A, N	Colour; static; M, F; adults	Implicit	Temporal–occipital sites	13.88	–	141.67	–
Dennis et al. (2009)	15	5−9	NimStim	Sa, F	B&W; static; M, F; adults	Implicit	O1, O2, Oz	15.11	–	135.22	–
Gu et al. (2019)	29	9−13	CFAPS	H, F, N	B&W; static; M, F; adults	Explicit	O1, O2	14.28	–	135.11 Measured at O1	–
Hum et al. (2013a)	34	8−12	NimStim^a^	H, A, C	Colour; static; M, F; adults	Implicit	O1	14.85	–	142.09	–
Hum et al. (2013b)	16	8−12	NimStim^a^	H, A, C	Colour; static; M, F; adults	Implicit	Occipital sites	14.62	–	138.91	–
Keil et al. (2018)	33	10−13	Karolinska	H, A, N	Colour; static; M, F; adults	Explicit	O1, O2	13.04	–	–	–
Luyster et al. (2017)	18	12	NimStim^b^	H, A, F, N	Colour; static; F; adults	Implicit	O1, O2	12.53	–	99.03	–
Meaux et al. (2014)	26	4−10	From Batty and Taylor (2003)	H, A, Sa, Su, F, D, N	B&W; static; M, F; adults	Implicit	O1, O2		P100 ↓*		N. S
Group 1	8	4−6						26.33		111.71	
Group 2	8	6−8						20.30		105.04	
Group 3	10	8−10						15.20		109.09	
Miki et al. (2011)	68	7−14	ATR^c^	H, N, A	B&W; dynamic; M, F; adults	Implicit	O1, O2		P100 ↓**		N. S
Group 1	39	7−10						6.68		135.64	
Group 2	29	11−14						3.12		132.15	
O’Connor et al. (2005)	15	9−15	MREL	H, A, Sa, F, N	B&W; static; M, F; children & adults	Explicit	O1, O2	5.59	–	142.25	–
Simonetti et al. (2019)	26	6−17	NIMH ChEFS, NimStim	H, F, N	B&W; static; M, F; children & adults	Implicit	O1, O2	9.27	–	114.85	–
Tye et al. (2014)	26	8−13	From Battaglia et al. (2004)	H[J], A, N, F, D	B&W; static; M, F; children	Implicit	O1, O2	9.69	–	141	–
Wong et al. (2008)	12	6−10	JACFEE	H, A, Sa, F, N	Colour; static; M, F; adults		O1, O2				–
						Implicit		11.02		116.56	
						Explicit		12.28		121.67	
Young et al. (2017)	37	12	NimStim^b^	H, A, F	Colour; static; M, F; adults	Implicit	Midline electrodes	10.05	–	120.68	–

Note. N.S = not significant - = not reported; ↓ = a decrease in P100 amplitude or latency with age. NimStim = NimStim set of facial expressions by Tottenham et al. (2009); POFA = Pictures of facial affect by Ekman and Friesen (1976); CFAPS = Chinese Facial Affective Picture System by Lu et al. (2005); KDEF = Karolinska directed emotional faces by Lundqvist et al. (1998); ATR = ATR Facial Expression Image Database by ATR Promotions; MREL = Mind Reading Emotions Library by Baron-Cohen et al. (2003); NIMH-ChEFS = National Institute of Mental Health Child Emotional Faces Picture Set by Egger et al. (2011); JACFEE = Japanese and Caucasian Facial Expressions of Emotion by (Matsumoto and Ekman, 1988). H = happy; J = joy; C = calm; A = anger; Sa = sad; Su = surprise; F = fear; D = disgust; N = neutral. B&W = Black and white; M = male, F = female.

^a^Stimuli included open and closed mouths; ^b^ stimuli presented at 20, 40 and 60 % intensities; ^c^ dynamic stimuli.

*p = <.05; **p = <.01; ***p = <.001.

#### 3.1.1 Methodological aspects of studies examining P100

Studies generally used a window between 90 and 150 ms post stimulus onset to detect the P100, with activity typically recorded across occipital electrodes (O1, O2). Studies with age ranges restricted to early childhood, such as D’Hondt et al. (2017), typically implemented a wider window (i.e., until 200 ms post stimulus onset), and tended to include temporal and/or parietal electrodes. There were differences in the average P100 amplitude between studies, ranging from 3.12 μV (Miki et al., 2011) to 27.5 μV (Batty et al., 2011). Sample size across studies ranged from 12 (Apicella et al., 2013) to 69 (Batty and Taylor, 2006), with a median of 26 participants.

Studies included a variety of stimulus sets, however the most commonly used was the NimStim set (Tottenham et al., 2009). Black and white stimuli were used for 12 studies, whilst the remaining seven studies used coloured images. Stimuli were mostly static (i.e., still images), however Miki et al. (2011) included dynamic (i.e., moving) stimuli. Luyster et al. (2017) and Young et al. (2017) used morphing software to present emotional stimuli at incremental emotional intensities up to 60 %. Of the basic emotions, each study included expressions of happiness, whilst expressions of surprise and disgust were included in the fewest studies. Three studies (Batty et al., 2011; Batty and Taylor, 2006; Meaux et al., 2014) included neutral stimuli and all six of the basic emotions. Most studies included adult stimuli, although Tye et al. (2014) used child stimuli, whilst O’Connor et al. (2005) and Simonetti et al. (2019) included both child and adult stimuli. Studies generally incorporated either implicit (*n* = 14) or explicit (*n* = 4) tasks, whilst Wong et al. (2008) included both. Typically, studies that incorporated explicit task designs included older participants and reported slower latencies and stronger amplitudes than implicit task designs.

### 3.2 P100 amplitude

#### 3.2.1 Meta-regression testing the influence of age on P100 amplitude

**Fig. 4:**
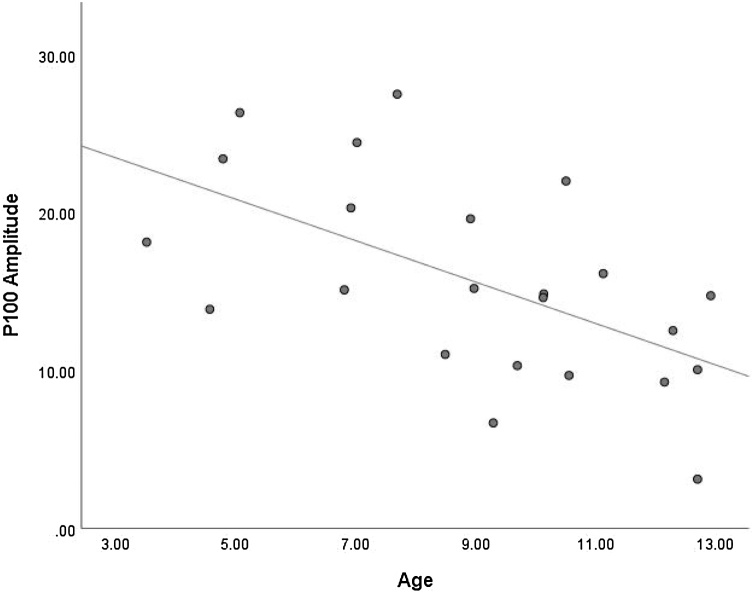
Scatterplot Showing the Relationship Between Age and P100 Amplitude.

Twenty-three datasets were included in a meta-regression to determine whether age is a predictor of P100 amplitude. Calculation of the *I*^2^ statistic revealed a value of 96.45, considered a high level of heterogeneity. This indicates that 96.45 % of the differences in P100 amplitude between studies reflect systematic influences. Results indicated that 33 % of between-study differences in P100 amplitude were accounted for by age, and this was statistically significant (*Q* (1, 22) = 8.85, *p* = .003, *R*^2^ = .33). As shown in Fig. 4, average P100 amplitude was typically smaller for studies in which the average age of children was larger. That is, P100 amplitude *decreased* with increasing age in children during implicit FEP tasks.

#### 3.2.2 Relationship between P100 amplitude and age

Five studies investigated the relationship between P100 amplitude and age during early-to-middle childhood (see Table 1). Four of the five studies reported significant age effects (Batty et al., 2011; Batty and Taylor, 2006; Meaux et al., 2014; and Miki et al., 2011). In line with the result of the meta-regression, all four studies reported decreases in P100 amplitude as age increased. The remaining study by Chronaki et al. (2018) did not find an influence of age upon P100 amplitude.

It is possible that the decrease in P100 amplitude might occur gradually throughout childhood. This is supported by Batty et al. (2011), who reported a significant reduction in P100 amplitude with increasing age across a sample aged between five and 15 years. If the reduction in amplitude is gradual, this may result in a lack of significant differences between adjacent age groups. Rather, differences may only become apparent when comparing the P100 amplitude between more disparate ages. In line with a gradually emerging P100 amplitude effect with age, Batty and Taylor (2006) found significant differences between all of their age group comparisons except when comparisons were between adjacent age groups. Similarly, Meaux et al. (2014) illustrated a reduction in P100 amplitude between younger (4−6 years) and older (8−10 years) age groups, but not for the middle (6−8 years) age group. Miki et al. (2011) found that P100 amplitude was significantly different between their adjacent age groups. The age groups in this study, however, used a wider range of four years (7−10 years and 11−14 years), which may help to explain this result.

Chronaki et al. (2018) did not find an age effect on P100 amplitude across their sample of five to 11-year-olds. Interestingly, this was the only study that examined age effects using an explicit task design. Thus, it may be that the increased attention required to complete an explicit task masked any underlying age effects. Together, these studies suggest a constant modification in P100 amplitude, with amplitude decreasing across increasing age groups spanning early-to-middle childhood.

### 3.3 P100 latency

#### 3.3.1 Meta-regression testing the influence of age on P100 latency

**Fig. 5:**
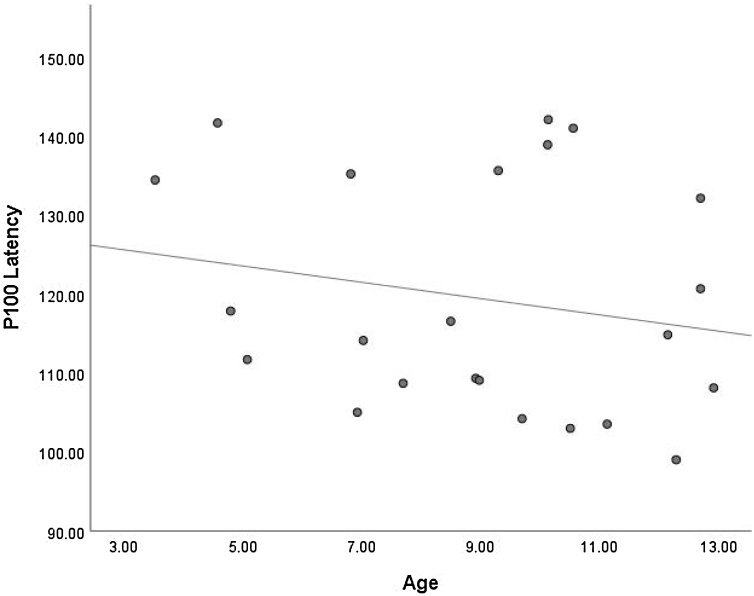
Scatterplot Showing the Relationship Between Age and P100 Latency.

Twenty-three datasets were included in a meta-regression to determine whether age is a predictor of P100 latency. Analyses yielded an *I*^2^ value of 97.30 %, considered a high level of heterogeneity. This indicates that 97.30 % of the variability in P100 latency between studies reflects systematic influences on the data. Results indicated that age was not a significant predictor of P100 latency, *Q* (1, 22) = 0.71, *p* = .401, *R*^2^ = .03. As highlighted below in Fig. 5, whilst there appears to be a slight tendency for P100 latency to decrease with age, this was not statistically significant. That is, P100 latency did not significantly change with increasing age in children during implicit FEP tasks.

#### 3.3.2 Relationship between P100 latency and age

Four studies investigated the relationship between P100 latency and age during early-to-middle childhood (see Table 1). In contrast to meta-regression results, Batty et al. (2011) and Batty and Taylor (2006) reported significant reductions in P100 latency with age. The remaining studies by Meaux et al. (2014) and Miki et al. (2011) reported no significant effects of age on the P100 latency.

Age effects reported by Batty and Taylor (2006) revealed that P100 latency in children in age groups 4−5 years, 6−7 years, and 8−9 years was significantly longer than P100 latencies in older children (aged 10–15 years). These results suggest that by the age of 10 years, children may have become more efficient at processing faces, and more broadly, visual information. This may help to explain the lack of significant age effects reported by Meaux et al. (2014). It is possible that in Meaux et al. (2014), the oldest age group of 8−10 years (mean age of 8.98 years) may not have developed the capability to process visual information as efficiently as the older children in Batty and Taylor (2006). A similar suggestion may also explain the lack of age effects reported by Miki et al. (2011). In that study, the young age group included children aged 7−10 years (mean age 9.3 years), and this group was compared to children aged 11−14 years (mean age 12.7 years). It is possible that the neural maturation achieved in both age groups were too advanced to detect meaningful differences in P100 latencies. That is, the variation across P100 latency findings might be reflective of the fact that Meaux et al. (2014) did not include an old enough sample, whilst Miki et al. (2011) did not include a young enough sample to sufficiently highlight age effects. This is also supported by significant results from Batty et al. (2011), which included a wide age range of children, between 5 and 15 years.

### 3.4 Summary of relationship between P100 and age

Overall, meta-regression indicated that age explained a significant proportion of between studies variance in P100 amplitude, but not in P100 latency. In other words, as age increased, P100 amplitude significantly decreased, but P100 latency did not significantly change in children during FEP tasks. From the results, it was evident that a substantial amount of variability between studies remained, even after accounting for age. Broadly, the majority of studies that analysed age effects found decreases in P100 amplitude with age, thereby supporting the meta-regression results. Findings are less clear for P100 latency, although it appears that there may be a very slight decrease with age, and it might be that by around 10 years of age a more substantial latency decrease has occurred. Overall, results indicate that FEP development is reflected across the P100 amplitude changes during early-to-middle childhood.

### 3.5 Development of N170

Twenty-five studies investigated the N170 ERP component. Table 2 summarises the methodology and results of all 25 studies. Six studies investigated the relationship between N170 and age during early-to-middle childhood. Below is an overview of methodological aspects of these studies and relevant findings.

**Table 2:** Methodological Summaries of Studies within the Review that Reported N170 Amplitude and/or N170 Latency.

Author	n	Age range	Facial emotion processing task design elements					N170 Amplitude		N170 Latency	
			Stimulus set	Emotions	Presentation	Task	Electrode sites	Recorded [μV]	Age effects	Recorded [ms]	Age effects
Apicella et al. (2013)	12	6−13	NimStim	H, F, N	B&W; static; M, F; adults	Implicit	T5, T6	−0.08	–	168.13	–
Battaglia et al. (2017)	200	6−14	From Battaglia et al. (2005)	H, A, N	B&W; static; 1 M & 1 F; children	Implicit	Cz	−11.31	–	115.18	–
Battaglia et al. (2007)	45	7−9	From Battaglia et al. (2005)	H[J], A, N	B&W; static; 1 M & 1 F; children	Implicit	Cz, C3, C4, Pz, Fz	−11.29	N. S	152.94	N. S
Batty et al. (2011)	30	4−15	From Batty and Taylor (2003)	H, A, Sa, Su, F, D, N	B&W; static; M, F; adults	Implicit	P7, P8				
Group 1	15	4−15						−2.94		195.64	
Group 2	15	5−15						−5.47		175.00	
Batty and Taylor (2006)	69	4−13	From Batty and Taylor (2003)	H, A, Sa, Su, F, D, N	B&W; static; M, F; adults	Implicit	P7, P8, TP9, TP10, PO9, PO10		N170 ↓*		N170 ↓***
Group 1	13	4−5						−4.81		216.75	
Group 2	15	6−7						−6.77		212.58	
Group 3	13	8−9						−5.80		189.39	Groups: 3 > 1*, 2*
											3 < 4*, 5*
Group 4	13	10−11						−4.09		151.37	
Group 5	15	12−13						−2.63		152.76	
Bertoletti et al. (2012)	47	8−10	From Battaglia et al. 2005	H[J], A, N	B&W; static; 1 M & 1 F; children	Implicit	Cz, C3, C4, Pz, Fz				
Group 1	35	8−10						−8.04	–	–	–
Group 2	12	8−10						−10.64	–	–	–
Chronaki et al. (2018)	58	5−11	POFA	H, A, N	Colour not stated; static; 2 F; adults	Explicit	Parietal & occipital sites	−9.37	N. S	233.2	–
Curtis and Cicchetti (2011)	25	3−4	POFA	H, A, N	B&W; static; 1 of 3 F; adults	Implicit	O1, O2, Oz	9.10 Measured at O1, O2	–	213.50 Measured at Oz	–
D’Hondt et al. (2017)	15	3−5	NimStim	H, A, N	Colour, static, M, F; adults	Implicit	Occipito-temporal sites	−0.30	–	219.00	–
Haan et al. (1998)	44	5	POFA	H, A, F	Colour not stated; static; 1 F; adult	Explicit	Fz, F3, F4, Pz, T5, T6	−0.63	–	169.29	–
Dennis et al. (2009)	15	5−9	NimStim	Sa, F, N	B&W; static; M, F; adults	Implicit	PO7, PO8, POz	11.48 For Sa & F	–	188.01	–
Grunewald et al. (2015)	26	11−14	NimStim	H, Sa, F, N[C]	B&W; static; gender not specified; adults	Explicit	P7, P8	−2.58	–	183.16	–
Gu et al. (2019)	29	9−13	CFAPS	H, F, N	B&W; static; M, F; adults	Explicit	P7, P8	−2.36	–	180.72 Measured at P8	–
Hoyniak et al. (2019)	26	3−5	NimStim	H, F	Colour not stated; static; F; adults	Implicit	Posterior electrodes	3.29	N170 ↑*	219.76	–
Hum et al. (2013a)	34	8−12	NimStim^a^	H, A, N[C]	Colour; static; M, F; adults	Implicit	Posterior electrodes	−1.18	–	–	–
Keil et al. (2018)	33	10−13	KDEF	H, A, N	Colour; static; M, F; adults	Explicit	P7, P8	1.16	–	–	–
Luyster et al. (2017)	18	12	NimStim^b^	N, H, A, F	Colour; static; F; adults	Implicit	Temporal-parietal sites	−1.22	–	156.6	–
Magnuson et al. (2020)	30	6−12	POFA	H, A	B&W; static; M, F; adults	Implicit	P3, P4	1.08	–	274.53	–
Meaux et al. (2014)	26	4−10	From Batty and Taylor (2003)	H, A, Sa, Su, F, D, N	B&W; static; M, F; adults	Implicit	T5, T6		N170 ↓*		N170 ↓**
Group 1	8	4−6						−10.11	Groups: 1 > 2*, 3*	227.30	Groups: 1 > 2*, 3*
Group 2	8	6−8						−3.85		200.66	
Group 3	10	8−10						−4.84		205.63	
Miki et al. (2011)	68	7−14	ATR^c^	H, A, N	B&W; dynamic; M, F; adults	Implicit	T5, T5′, T6, T6′		N. S		N. S
Group 1	39	7−10						−8.16		236.07	
Group 2	29	11−14						−7.25		222.93	
O’Connor et al. (2005)	15	9−15	MREL	H, A, Sa, F, N	B&W; static; M, F; adults & children	Explicit	P3, P4	−4.32	–	217.57	–
O'Toole et al. (2013)	27	5−7	NimStim	H, A, N	B&W; static; M, F; adults	Implicit	P5, P6, P7, P8	−0.39	–	211.61	–
Tye et al. (2014)	26	8−13	From Battaglia et al. 2004	H[J], A, F, D, N	B&W; static; M, F; children	Implicit	P7, P8	−6.28	–	207.00	–
Wong et al. (2008))	12	6−10	JACFEE	H, A, Sa, F, N	Colour; static; M, F; adults		T5, T6		–		–
						Implicit		−3.66		184.62	
						Explicit		−5.56		191.03	
Young et al. (2017)	37	12	NimStim^b^	N, H, A, F	Colour; static; M, F; adults	Implicit	T6	−0.55	–	165.37	–

Note: NS = not significant; - = not reported; ↑ = an increase (i.e., more negative N170 amplitude) with age; ↓ = a decrease (i.e., less negative N170 amplitude) or shorter N170 latency with age. NimStim = NimStim set of facial expressions by Tottenham et al. (2009); POFA = Pictures of facial affect by Ekman and Friesen (1976); CFAPS = Chinese Facial Affective Picture System by Lu et al. (2005); KDEF = Karolinska directed emotional faces by Lundqvist et al. (1998); ATR = ATR Facial Expression Image Database by ATR Promotions; MREL = Mind Reading Emotions Library by Baron-Cohen et al. (2003); NIMH-ChEFS = National Institute of Mental Health Child Emotional Faces Picture Set by Egger et al. (2011); JACFEE = Japanese and Caucasian Facial Expressions of Emotion by Matsumoto and Ekman (1988) . H = happy; J = joy; C = calm; A = anger; Sa = sad; Su = surprise; F = fear; D = disgust; N = neutral. B&W = Black and white; M = male, F = female.

^a^ Stimuli included open and closed mouths; ^b^ stimuli presented at 20, 40 and 60 % intensities; ^c^ dynamic stimuli.

*p=<.05; **p=<.01; ***p=<.001.

#### 3.5.1 Methodological aspects of studies examining N170

Detection of the N170 was typically within timeframes of 140−240 ms, however Battaglia et al. (2017) reported a latency of 115.18 ms. As expected, there was a tendency for studies including younger samples, such as Curtis and Cicchetti (2011), to record wider N170 time windows (up to 300 ms). Examining N170 activity was primarily achieved by utilizing posterior temporal electrodes (T5, T6, P7, P8), however Battaglia et al. (2017) recorded N170 using Cz. The selected electrodes seemed dependent upon the age of the sample. Studies with younger children, such as D'Hondt et al. (2017), typically utilised occipito-temporal electrode regions, whilst studies focusing purely on activity in older children (Grunewald et al., 2015; Young et al., 2017) primarily recorded from temporo-parietal sites. Differences in the average amplitudes recorded ranged from -11.44 μV (Battaglia et al., 2007) to 11.48 μV (Dennis et al., 2009). Sample size ranged from 12 (Wong et al., 2008) to 200 (Battaglia et al., 2017), with a median of 29 participants.

A range of stimulus sets were used, however the most common were coloured, static adult male and female stimuli from the NimStim (Tottenham et al., 2009). Studies by Battaglia et al. (2017), 2007; Bertoletti et al. (2012), and Tye et al. (2014) included child stimuli adopted from previous work by Battaglia et al. (2005), whilst O’Connor et al. (2005) included both adult and child stimuli. Luyster et al. (2017) and Young et al. (2017) used morphing software to present emotional stimuli at incremental emotional intensities up to 60 %. All studies except Dennis et al. (2009) included expressions of happiness or joy, whilst three studies (Batty et al., 2011; Batty and Taylor, 2006; Meaux et al., 2014) included all six basic emotions, in addition to neutral stimuli. Studies incorporated either implicit (*n* = 18) or explicit (*n* = 5) emotion processing tasks, with Wong et al. (2008) including both. Task design included primarily implicit tasks for younger samples, whilst studies exploring older samples, such as Keil et al. (2018) and Gu et al. (2019), tended to incorporate explicit tasks. Generally, explicit task designs were associated with larger latencies and stronger negative amplitudes than implicit tasks.

### 3.6 N170 amplitude

#### 3.6.1 Meta-regression testing the influence of age on N170 amplitude

**Fig. 6:**
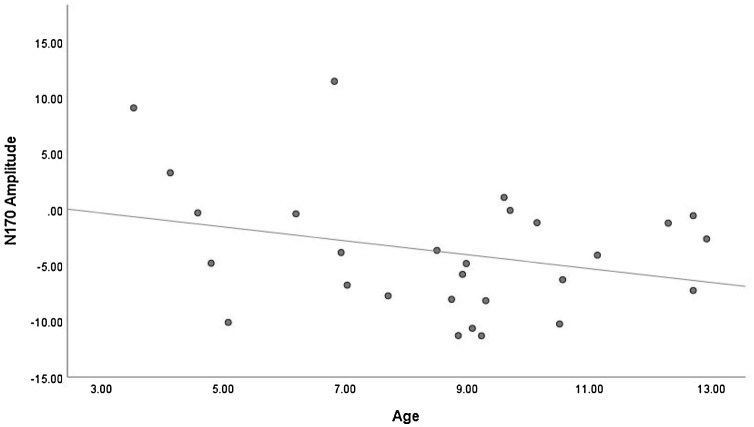
Scatterplot Showing the Relationship Between Age and N170 Amplitude.

Twenty-eight datasets were included in a meta-regression to determine whether age is a predictor of N170 amplitude. Analyses yielded an *I*^2^ value of 97.64 %, considered a high level of heterogeneity. This indicates that 97.64 % of the variability in N170 amplitude between studies reflects systematic influences on the data. Meta-regression indicated that age was not a significant predictor of N170 amplitude (*Q* (1, 27) = 2.94, *p* = .086, *R*^2^ = .09). As highlighted in Fig. 6, while there appears to be a weak trend for N170 amplitude to become stronger (i.e., more negative) with age, this trend is variable. Overall, N170 amplitude did not significantly change with increasing age in children during implicit FEP tasks.

#### 3.6.2 Relationship between N170 amplitude and age

Six studies investigated the relationship between N170 amplitude and age during early-to-middle childhood (see Table 2). Three studies reported significant age effects, however two studies reported that N170 amplitude became weaker (i.e., less negative) with increasing age (Batty and Taylor, 2006; Meaux et al.;, 2014), while one study found N170 amplitude became stronger (i.e., more negative) with age (Hoyniak et al., 2019). The remaining three studies (Battaglia et al., 2007; Chronaki et al., 2018; Miki et al., 2011) reported no meaningful changes in N170 amplitude with age.

It is possible that age influences N170 amplitude in a non-linear manner. The study by Hoyniak et al. (2019) with the youngest age group of children aged three to five years found significant age effects, whereby the N170 became larger with age. Rather than continuing to become larger with age, studies with older children sometimes found effects in the opposite direction. Indeed, Meaux et al. (2014) reported that their age group of four to six years had a larger amplitude than both the six to eight and eight to 10-year age groups, with no difference between the older two groups. That is, N170 amplitude became smaller at around four to eight years of age, with no substantial change after that. Batty and Taylor (2006) also found a decrease in N170 amplitude with age, though only tested this across their whole sample of four to 12 years. While statistical analysis between age subgroups was not undertaken, it appears that amplitude may have initially increased between the four to five years and six to seven years groups before decreasing from seven to 12 years. Studies that included children with a mean age between eight and 12 years found non-significant effects of age on N170 amplitude (Battaglia et al., 2007; Chronaki et al., 2018; Miki et al., 2011). This may indicate that fewer changes are occurring in N170 amplitude during middle childhood. Taken together, these data suggest an initial increase in N170 amplitude in very young children, followed by a decrease that becomes smaller into later childhood.

Differences in stimuli and task design were observed in the three studies that reported no meaningful changes. For example, the presentation of dynamic stimuli used by Miki et al. (2011), child stimuli used by Battaglia et al. (2007), and the implementation of an explicit task design used by Chronaki et al. (2018) may have influenced N170 amplitudes. Therefore, it is possible that the presence of age differences may have been masked by methodological differences. Alternatively, and in support of the meta-regression results, it is possible that N170 amplitude does not significantly change with age in children during implicit FEP tasks.

### 3.7 N170 latency

#### 3.7.1 Meta-regression testing the influence of age on N170 latency

**Fig. 7:**
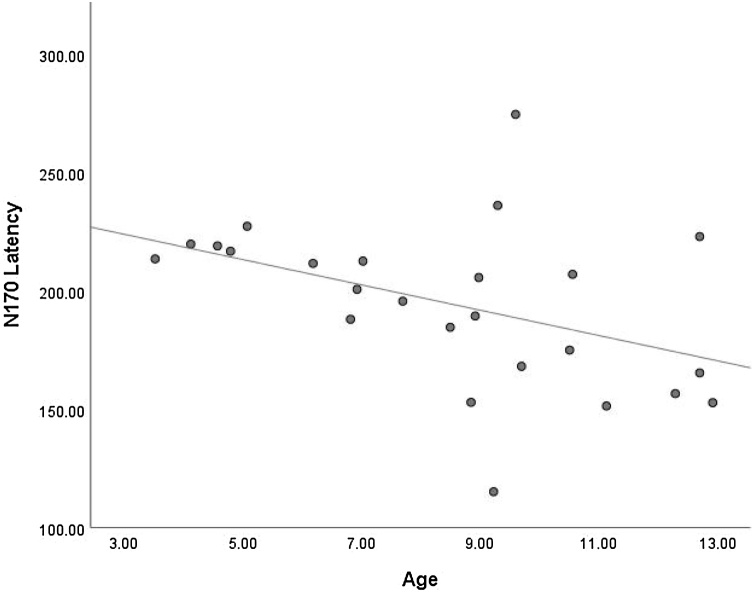
Scatterplot Showing the Relationship Between Age and N170 Latency.

Twenty-five datasets were included in a meta-regression to determine whether age is a predictor of N170 latency. Analyses yielded an *I*^2^ value of 99.48, considered a high level of heterogeneity. This indicates that 99.48 % of the variability in N170 latency between studies reflects systematic influences on the data. Meta-regression indicated that age was not a significant predictor of N170 latency (*Q* (1, 24) = 2.66, *p* = .103, *R*^2^ = .19). As highlighted in Fig. 7, while there appears to be a trend for shorter N170 latencies with increasing age, this trend is variable, particularly around the ages of nine to 10 years. Overall, N170 latency did not significantly change with increasing age in children during implicit FEP tasks.

#### 3.7.2 Relationship between N170 latency and age

Four studies explored the effect of age on N170 latency across early-to-middle childhood (see Table 2). Of these studies, Batty and Taylor (2006), and Meaux et al. (2014) reported reductions in latency with age, whilst Battaglia et al. (2007) and Miki et al. (2011) reported no age effects. Results by Miki et al. (2011), however, revealed significant decreases in N170 latency when comparing young and old children to adults.

Exploration of results revealed that the statistically significant reductions were not consistent with age across early-to-middle childhood. Indeed, each of the studies that reported significant age effects included participants in early childhood (Batty and Taylor, 2006; Meaux et al., 2014). However, the ages at which N170 latency markedly reduced differed across these studies. Analyses by Meaux et al. (2014) revealed that the statistically significant effect was present when comparing the 4−6 age group with both the 6−8 years and 8−10-year age groups, but not between the older two age groups. Conversely, for Batty and Taylor (2006), statistically significant differences in N170 latencies were only reported between the age group of 8−9 years, when compared to all other ages. Therefore, these results suggest that the main age for change in N170 latency may lie around the ages of the transition between early and middle childhood, thus highlighting the importance of this developmental period.

Both of the studies that included a sample without early childhood participants (Battaglia et al., 2007; Miki et al., 2011) failed to report significant age effects. It is plausible that the age of participants across these studies were too old to reflect any meaningful differences in N170 latencies. This may explain the lack of significant differences in N170 latency between the younger age group (mean of 9.3 years) and older age group (mean of 12.7 years) in the study by Miki et al. (2011). Whilst Miki et al. (2011) included an age range of seven to 14 years, the age of participants was negatively skewed. Furthermore, it is possible that the age range (7−9 years) included by Battaglia et al. (2007) was too narrow to detect any age effects.

The influence of stimuli presentation may help to explain differences across recorded latencies when comparing similarly aged children. For example, Battaglia et al. (2017) reported an overall average N170 latency of 115 ms for children with a mean age of 9.23 years, whilst Miki et al. (2011) recorded an average latency of 245 ms amongst children with a mean age of 9.30 years. Although Miki et al. (2011) reported a latency more than twice that of Battaglia et al. (2017), important methodological differences may assist in explaining this variation. Miki et al. (2011) presented stimuli that were morphed from a neutral expression to an affective expression, thereby mimicking dynamic facial movement, whilst Battaglia et al. (2017) displayed static facial stimuli. It is therefore plausible that the longer N170 latencies recorded by Miki et al. (2011) may reflect variation in the cognitive and visual processors required to interpret an actively forming facial expression, thereby raising issues of comparability. This seems to suggest that some of the variation across the FEP related neural activity may highlight stimulus presentation differences, rather than suggest an inconsistency of N170 latency for similarly aged children.

Age effects were present only in the studies that included particular emotions. Both studies that reported age effects (Batty and Taylor, 2006; Meaux et al., 2014) included emotions such as fear, disgust, sadness, and surprise, which are known to develop later in childhood (Lawrence et al., 2015). This highlights the possibility that age effects of the N170 latency may be observable in more complex emotions that are continuing to emerge during middle childhood, whilst age effects may have already plateaued for emotions such as happiness and anger at an earlier age. Of the studies that reported no age effects (Battaglia et al., 2007; Miki et al., 2011), emotional stimuli were restricted to a version of happiness (or joy), anger, and neutrality. Coupled with the older age of participants, it is possible that the emotional stimuli selected in these studies may have hindered the presence of any age effects.

### 3.8 Summary of relationship between N170 and age

Overall, meta-regression indicated that age did not explain a significant proportion of between studies variance in either the N170 amplitude, or the N170 latency. In other words, as age increased throughout early-to-middle childhood, N170 amplitude and N170 latency did not significantly change. However, both amplitude and latency did show non-significant trends, with N170 amplitude tending to become stronger with increasing age, whilst N170 latency tended to decrease with increasing age. This could indicate that a lack of power and lack of precision within individual studies may be contributing to the non-significant result. It was also evident that a substantial amount of variability between studies remained even after considering age. Examination of the studies that investigated age effects revealed mixed support for the meta-regression findings. Results by Meaux et al. (2014) and Batty and Taylor (2006) contrasted with the meta-regression trend towards the N170 amplitude becoming stronger with age. This may be due to the age of participants, or the seemingly non-linear pattern of development of the N170 amplitude. Indeed, this non-linear trend may help to explain why the meta-regression was not significant. For N170 latency, the studies that analysed age effects broadly supported the trend in Fig. 7, with a decreasing latency alongside increasing age, although this was not significant.

### 3.9 Development of late positive potential

Eleven articles explored the Late Positive Potential (LPP) ERP component (see Table 3). Unlike the P100 and N170, the LPP is measured by the average voltage across a wide time period. As stated earlier, a meta-regression was not undertaken due to the variability in measurement of the LPP. Below is an overview of methodological aspects of these studies and relevant findings.

**Table 3:** Methodological Summaries of Studies (and subgroups) within the Review that Reported LPP Amplitude and Age Effects.

Author	n	Age range	Facial emotion processing task design elements					Mean LPP amplitude		
			Stimulus set	Emotions	Stimulus presentation	Task	Electrode sites	Definition [ms post onset]	Recorded [μV]	Age effects
Burkhouse et al. (2019)	58	7−11	NimStim^b^	F, H, Sa, N	Colour; dynamic; M, F; adults	Explicit	O1, O2, Oz, P3, P4, PO3, PO4, Pz	400−1000	DNS	–
Chronaki et al. (2018)	58	6−11	POFA	H, A, N	Not stated; static; 2 F; adults	Explicit	Occipital sites	Early: 430−520	Early: 9.35 Late: 3.70	–
								Late: 520−610		
							Parietal sites		Early: 9.63	Early LPP ↑ for H, N**
									Late: 8.69	Late LPP ↑ for H, N**
Connell et al. (2019)	48	10−14	NimStim	H, Sa, C	Not stated; static; M, F; adults	Explicit	P3, P4, Pz	500−700	10.54	–
Grunewald et al. (2019)	26	11−14	NimStim	H, Sa, F, C	B&W; static; gender not specified; adults	Explicit	Pz, P3, P4, CP1, CP2, Cz, C4	400−1500	3.70	–
Gu et al. (2019)	29	9−13	CFAPS	H, F, N	B&W; static; M, F; adults	Explicit	C3, C4, Cz, P3, P4, Pz	400−800	11.37	–
James et al. (2018)	87	7−11	NIMH-ChEFS^b^	F, H, Sa, N	B&W; dynamic; M, F; children	Explicit	O1, O2, Oz, P3, P4, PO3, PO4, Pz	400−1000		–
Group 1	63	7−11							0.84	
Group 2	24	7−11							−2.02	
Keil et al. (2018)	33	10−13	KDEF	H, A, N	Colour; static; M, F; adults	Explicit	O1, O2, PO3, PO7, PO4, PO8	Early: 300−600	Early: 13.36	Middle LPP↓ for H*
								Middle: 600−1000	Middle: 12.94	Late LPP↓ for H, N*
								Late: 1000−2000	Late: 9.57	
Kujawa, Hajcak et al. (2012)	188	5−7	NimStim	Sa, H, A, F, N	Colour not stated; static; M, F; adults	Implicit	O1, O2, Oz, P3, P4, Pz	Early: 200−600	DNS	–
								Late: 600−1000		
Kujawa, Klein, Hajcak (2012)	58	8−13	KDEF	H, N, Sa	Colour not stated; static; M, F; adults	Implicit	Occipital: O1, O2, Oz	400−1000		Parietal LPP ↑*
							Parietal: P3, P4, Pz			Occipital LPP NS
Group 1	DNS	8−10							Occipital: 21.39	
									Parietal: 1.01	
Group 2	DNS	11−13							Occipital: 18.72	
									Parietal: 4.42	
Simonetti et al. (2019)	26	6−17	NIMH ChEFS & NimStim	H, F, N	B&W; static; M, F; children & adults	Implicit	O1, O2, Oz	400−1000	3.81	–
Woody et al. (2019)	37	7−11	NIMH-ChEFS^a^	H, A, F, N	B&W; morphed; M, F; children	Explicit	O1, O2, Oz, P3, P4, PO3, PO4, Pz	400−1000	Low: 12.86	–
									Medium: 13.53	
									High: 14.71	

Note: D.N.S = does not state; - = not reported; ↑ = an increase in LPP amplitude with age; ↓ = a decrease in LPP amplitude with age. NimStim = NimStim set of facial expressions by Tottenham et al. (2009); POFA = Pictures of facial affect by Ekman and Friesen (1976); CFAPS = Chinese Facial Affective Picture System by Lu et al. (2005); KDEF = Karolinska directed emotional faces by Lundqvist et al. (1998); NIMH-ChEFS = National Institute of Mental Health Child Emotional Faces Picture Set by Egger et al. (2011). H = happy; C = calm; A = anger; Sa = sad; F = fear; N = neutral. B&W = Black and white; M = male, F = female.

^a^ stimuli presented at 20, 40 and 60 % intensities; ^b^ dynamic stimuli.

*= <.05; ** = <.01.

#### 3.9.1 Methodological aspects

Across studies, the LPP was typically identified between 400–1500 ms post stimulus onset. Five studies used the average amplitude within a window of 400−1000 ms. Other studies split the time window into narrower widths allowing comparison of the LPP at early, middle, and late stages. Nevertheless, differences in defining the time window of the LPP were evident, with maximum timepoints of “late” LPP time windows ranging from 520 ms (Chronaki et al., 2018) to 2000 ms (Keil et al., 2018). Typically, LPP amplitude was recorded across a combination of parietal and occipital sites, with two studies reporting the LPP amplitudes separately across these sites. Differences in the average LPP amplitudes recorded ranged from -2.02 μV (Battaglia et al., 2007) to 21.39 μV (Kujawa et al., 2012a, 2012b). Typically, LPP amplitude was larger for early windows, when compared to late windows. This is likely reflective of the fact that the LPP wave initially increases in amplitude before tapering towards zero.

Sample size across studies ranged from 26 (Grunewald et al., 2019) to 188 (Kujawa, Hajcak et al., 2012), with a median of 48 participants. Most studies employed an explicit task design, with the exception of Kujawa et al. (2012a, 2012b) and Simonetti et al. (2019). Generally, adult stimuli were used in the FEP task, although James et al. (2018) and Woody et al. (2019) included morphed child stimuli. The majority of studies included children aged seven years or older, with no studies including children under 5 years of age. Thus, unlike the P100 and N170, it is impossible to explore the LPP amplitude across the entire early-to-middle childhood range.

### 3.10 LPP amplitude

#### 3.10.1 Relationship between LPP amplitude and age

Studies by Chronaki et al. (2018); Kujawa et al. (2012a, 2012b) and Keil et al. (2018) explored age effects on the LPP amplitude. However, important differences between these studies made it difficult to directly compare results. For example, one study split the LPP into narrower widths (Keil et al., 2018), another recorded LPP amplitude separately across parietal and occipital sites (Kujawa et al., 2012a, 2012b), and the third study both split the LPP into narrower widths and recorded LPP amplitude separately across parietal and occipital sites (Chronaki et al., 2018). The two studies that recorded separate LPPs for occipital and parietal sites found that LPP amplitude increased with age for parietal sites only (Chronaki et al., 2018; Kujawa, Klein, et al., 2012). Chronaki et al. (2018) found this was the case for both the early and late windows, whereas Kujawa et al. (2012a, 2012b) recorded only a single time window. Keil et al. (2018), using the average of parietal and occipital channels, found age effects (for their middle and late windows), in the opposite direction, whereby LPP amplitude decreased with age.

There were large differences in defining the time window of average, early, middle or late LPP amplitudes. For example, the early window investigated by Keil et al. (2018) almost spans both the early and late windows used by Chronaki et al. (2018). This suggests that the activity recorded during these ‘defined’ early and late time frames may not be comparable. As Chronaki et al. (2018) did not include an LPP time window past 610 ms, it is possible that a different pattern of neural activity may be present in the later period, although this was not examined. Furthermore, since Kujawa et al. (2012a, 2012b) included an overall average value of LPP activity, it is possible that specific changes in LPP amplitude with age are occurring in smaller windows during their 400−1000 ms timeframe that may provide additional developmental FEP information.

In line with previous research indicating that the LPP amplitude may be influenced by the elaborated meaning of facial stimuli (see Hajcak et al., 2009), LPP age effects tended to be emotion specific. Both Chronaki et al. (2018) and Keil et al. (2018) reported age effects for happy and neutral but not angry stimuli, although the direction of the reported age effects were opposite. Early (430−520 ms) and late (520−610 ms) parietal LPP amplitudes were found by Chronaki et al. (2018) to increase with age for happy and neutral stimuli. Keil et al. (2018) reported that the middle (600−1000 ms) LPP amplitude significantly decreased with age for only happy stimuli, whilst the later (1000−2000 ms) LPP significantly decreased with age for both happy and neutral stimuli. These emotion-dependent differences are contrasted Kujawa et al. (2012a, 2012b), who reported an increase in LPP amplitude with no emotion-specific differences across parietal sites for their happy, neutral, and sad stimuli.

In explaining the contrasting age effects, it is possible that the development of the LPP follows a non-linear trajectory. It is theoretically conceivable that LPP amplitude may initially increase with age, before decreasing at the commencement of adolescence. This would support Chronaki et al. (2018), who reported positive associations between age and amplitude across children with a mean age of 8.8 years, and Kujawa et al. (2012a, 2012b) who found an increase from 8−10 to 11−13-year olds. Furthermore, this may indicate why Keil et al. (2018) reported reductions in LPP amplitude across children aged 10−13 years.

#### 3.10.2 Summary of relationship between LPP amplitude and age

In summary, although results were minimal and mixed, the literature suggests that LPP amplitude undergoes significant changes throughout early-to-middle childhood with reference to facial emotional stimuli. Overall, the relationship between LPP amplitude and age indicates emotion-specific differences, however, it remains uncertain whether LPP development follows a linear or non-linear trajectory across childhood.

## 4 Discussion

The main aim of this review was to chart the developmental course of the electrophysiological (EEG) response to emotional faces throughout early-to-middle childhood (3–12 years), through examining P100, N170, and LPP. In support of the hypothesis, results from both meta-regression and individual studies indicate that P100 amplitude decreases with age. In contrast with the hypothesis, meta-regression indicated that P100 latency does not change with age. However, a review of the literature suggests a possible decrease in latency at roughly 10 years old. In line with the hypothesis, meta-regression indicated a trend towards a larger, more negative N170 amplitude with age, however this did not reach statistical significance. Review of individual studies suggested that N170 amplitude may change with age in a non-linear manner. In opposition with the hypothesis, N170 latency did not decrease with age. Examination of individual studies suggest that N170 latency might decrease more rapidly in early childhood, with only studies including younger participants reporting age effects. Finally, it was hypothesised that LPP amplitude would decrease with age. Whilst meta-regression could not be undertaken, an overall summary of existing literature revealed some evidence to suggest that development of the LPP amplitude has emotion-specific age effects.

This review builds upon a previous review of the literature by Taylor et al. (2004), by extending facial processing to include the processing of facial emotions. Meta-regression results support Taylor et al. (2004), indicating that P100 amplitude significantly decreases with increasing age during early-to-middle childhood across implicit FEP tasks. It is possible that this reduction in amplitude is reflective of a gradually emerging specialisation in face processing during childhood. Meta-regression results indicated that P100 latency did not decrease with increasing age during early-to-middle childhood, thus contesting the results from Taylor et al. (2004). Whilst non-significant, a slight downward trend in P100 latency with increasing age appeared to be steepest during early childhood. Broadly, this development of the P100 might be indicative of an increasing efficiency in the processing of expressive visual facial information due to synaptic pruning.

Meta-regression did not support the hypothesis that N170 amplitude would increase (i.e., become more negative) with age. One reason for this may be that the development across early-to-middle childhood is non-linear. Inspection of Fig. 6 suggests a non-linear relationship between age and N170 amplitude. Close examination of individual studies suggest that N170 amplitude might display an initial steep increase in early childhood, before decreasing in amplitude in middle childhood. The initial increase in N170 amplitude in early childhood may reflect the activity of two separate but increasingly overlapping neural sources considered precursors to the N170. It is possible that the merging of these precursors may be reflected in a decrease in N170 amplitude during the ages of 8–12 years. Indeed, this is in line with previous suggestions that the merging of these precursors occurs during latter stages of middle childhood (Batty and Taylor, 2006; Taylor et al., 2004). This possible merging of precursors to the N170 could be arguably indicative of an increasing specialisation of emotional face processing networks.

Meta-regression results for age and N170 amplitude contest previous findings by Taylor et al. (2004) who reported a decrease in N170 amplitude (i.e., becoming less negative) with age. It is possible that the processing of *expressive* facial emotions as recorded by N170 amplitude, may display a distinctly different relationship with age, as compared to *neutral* facial processing explored by Taylor et al. (2004). Indeed, previous literature has suggested that the N170 is sensitive to emotion, with larger amplitudes for negative expressions (Batty and Taylor, 2006; Hinojosa et al., 2015). As the ability to distinguish between different emotions improves across childhood, this emotion-specific development is likely also reflected in the N170 development (Lawrence et al., 2015).

Meta-regression did not support the hypothesis that N170 latency would decrease with age. It may be that the development across early-to-middle childhood is not consistent. Inspection of Fig. 7 seems to indicate an inconsistent relationship between age and N170 latency. Examination of individual studies suggests that there is a steady decrease in N170 latency up to the age of around eight years old, indicating increasing efficiency in the processing of expressive facial stimuli. This pattern appears to continue in middle childhood, however at a slower and more variable rate. It is possible that decreases in N170 latency may reflect ongoing reductions in cognitive effort required during FEP.

Meta-regression results for age and N170 latency contrast with previous findings by Taylor et al. (2004). Taylor et al. (2004) reported a steady decrease in N170 latency across the ages of four to 11 years, with the steepest decline occurring between the ages of eight and 10 years. However, the current review suggests that the age of greatest change may be younger, at around six to eight years. It is possible that the age of greatest change may be earlier in childhood for the processing of expressive facial stimuli, when compared to the processing of neutral facial stimuli. As expressive stimuli included only ‘basic’ emotions, it is possible that age effects of the N170 latency may be present in middle childhood for more complex emotions such as embarrassment or shame.

Findings suggest that LPP development may be modulated by emotion. In relation to LPP amplitude, Chronaki et al. (2018) and Keil et al. (2018) reported significant (yet opposing) age effects for happiness and neutrality, but not expressions of anger. This may indicate variation in the processing of positively and negatively valenced stimuli across childhood, or in the development of this FEP ERP. From an evolutionary perspective, is possible that the processing of negative emotional stimuli, such as anger, may continue to elicit strong recorded activity regardless of age. Consequently, it is possible that the processing of positive stimuli which provide fewer adaptive advantages, may decrease with age alongside heightened neural specialisation. Alternatively, the processing of threatening faces may develop during infancy and early childhood, and therefore may not display age effects across middle childhood. Nevertheless, there appeared to be possible emotion specific effects linked to the LPP, although this is speculative due to the limited number of studies reviewed, and therefore requires further research.

It is important to consider how the findings in this review relate to the wider literature and theoretical framework. Whilst findings from this review suggest that FEP development is reflected in changes in the P100, this does not necessarily demonstrate that the P100 is a face sensitive component. Indeed, this is supported in previous research showing that age-related changes in P100 activity followed similar patterns with response to facial and non-facial stimuli (Kuefner et al., 2010). Rather, findings may be suggestive of gradual structural and functional changes in general visual processing and encoding, as opposed to a gradual specialisation in specifically expressive FEP (Meaux et al., 2014; Miki et al., 2011). Contrastingly, the findings of this review in relation to the N170 likely provide greater insight into the development of FEP ERPs. Instead of a gradual decrease in amplitude (as the P100 demonstrated), the N170 amplitude appears to demonstrate a more complex relationship between FEP and age. The initial increase in N170 amplitude in early childhood may reflect the development of face-related regions, including the fusiform face area (FFA) and posterior superior temporal sulcus (pSTS), believed to be the source of the N170 (Gao et al., 2019). Indeed, it is possible that the following decrease in N170 amplitude into later childhood may illustrate the transformation from featural to configural processing of facial emotions (Aylward et al., 2005).

Substantial methodological differences across the studies in this review, as reflected in the high values of heterogeneity attributable to systematic influences, highlighted limitations in synthesising the literature. Despite extensive exclusion criteria, and acknowledging inter-individual variability, it is likely that methodological differences in the studies included in this review may have influenced the results. Indeed, we acknowledge that it is possible that results may be reflective of methodological differences. Previous research has suggested that there may be an own-age bias in the neural processing of facial stimuli (Wiese et al., 2008). For example, Melinder et al. (2010) demonstrated that N170 amplitudes in five-year-olds were significantly larger for faces of children than either adults or elderly adults. As studies in this review included adult, child, and both adult and child stimuli, it is possible that the existence of an own age bias may have influenced the results of this review. Furthermore, previous research has suggested that dynamic stimuli, and stimuli with stronger intensities of expressions often elicit longer latencies and larger amplitudes when compared to that of static or less intense stimuli (Jiang et al., 2014; Luyster et al., 2019; Sprengelmeyer and Jentzsch, 2006). As studies in this review included a variety of dynamic, static and morphed stimuli across a range of emotional intensities it is possible that the choice of stimuli influenced the results of this review. Therefore, it is likely that meta-regression results are not only measuring average age effects, but also illustrate the influence that task demands (cognitive load on processing static vs. dynamic or morphed faces), and the content of the stimuli (emotions expressed, age of stimuli model) have upon FEP ERPs. Moreover, it is important to acknowledge that underlying individual differences that may have influenced FEP development (Burkhouse et al., 2019). For example, previous research has demonstrated associations between distinct neural FEP ERP activity and individual differences in levels of anxiety (Chronaki et al., 2018; O’Toole et al., 2013) and genetics (Battaglia et al., 2007, 2017). It is recommended that future research reviews how individual differences may influence the development of FEP ERPs.

This review highlights the lack of research using narrow age groups within a wider sample conducted around the transitional period from early-to-middle childhood. Further accentuating the importance of this gap are coinciding social and environmental changes that occur alongside the commencement of primary school. Although not surprising, issues with sample size, risk of bias, and power permeate the studies in this review. With half of all the studies including a sample size of 26 participants or less, this review highlights the need for conducting large-scale collaborative studies. In terms of risk of bias, the majority of studies failed to report the extent of missing or excluded outcome data, whilst nearly half of the studies were assessed as having a definitely high or a probable high risk of selective reporting bias. This highlights the need for transparency across the reporting of data analyses and results. Additionally, due to variation across methodologies, publication bias was unable to be assessed, however the impact of publication bias cannot be disregarded.

Of the studies included in this review, only Woody et al. (2018) reported consistently adequate power across analyses. As discussed by Button et al. (2013), small sample sizes that are lacking power may prevent the detection of true effects. Given that research within developmental neurocognitive psychology is interested in detecting small but meaningful age effects on FEP ERPs, it is possible that the plethora of small sample sizes used may lack the power needed to detect the presence of small, yet true effects. Additionally, one must acknowledge the existing limitations when conducting a systematic review with meta-regression analyses. As several of the ERP component standard error values were not reported, values had to be estimated using the median across comparable samples. The non-significant findings coupled with visible trends, imply that a lack of power extended to the meta-regression analyses as well. Additionally, the lack of literature measuring the LPP in early-to-middle childhood prevented a meta-regression from being conducted. Since many studies included in this review reported values averaged across emotions, rather than values reflecting the difference between neutral and emotional faces, the use of ERP values for the average of emotional and neutral facial stimuli was necessary. Therefore, it is acknowledged that the inclusion of neutral stimuli in this review limits findings, as results are not explicitly reflective of expressive emotional facial stimuli. Nevertheless, there are interesting differences when comparing findings from this review to previous work by Taylor et al. (2004) who focused solely on neutral facial stimuli. In sum, this review stresses the need for emotion-specific reporting of FEP ERP values in large samples across early-to-middle childhood to enable a greater understanding of expressive FEP development.

This review provides a comprehensive analysis into FEP during early-to-middle childhood. Results from the P100 amplitude show that initial processing of facial configuration becomes more specialised and efficient across childhood. However, it is possible results are reflective of general visual processing. Findings from this review suggest that the development of N170 and LPP may be modulated by facial expressions, though additional research is required to determine the extent of this sensitivity. Furthermore, this review suggests that expressive facial processing may follow a different developmental trajectory to that of neutral facial processing across children aged three to twelve years. Consequently, this has potential implications for our understanding of social cognitive development. Future work may consider the development of more complex facial emotions during early-to-middle childhood and explore how this may relate to individual emotional and social cognitive skills.

## Data availability statement

The data that support the findings of this study are available in the supplementary material.

## Declaration of Competing Interest

None
