# Supplementary material for: The development of neural responses to emotional faces: A review of evidence from event-related potentials during early and middle childhood
Source: Dev Cogn Neurosci. 2021 Jul 21;51:100992. doi: 10.1016/j.dcn.2021.100992 (PMC8339225; doi:10.1016/j.dcn.2021.100992)
Supplement: Supplementary file 1 [file mmc1.docx]

**Supplementary material**

**Risk of Bias**

Risk of bias in individual studies was assessed using the NTP-OHAT Risk of Bias Assessment Tool (National Institute of Environmental Health Sciences, 2015). Figure S1 shows the NTP-OHAT risk of bias assessment across studies. Statistical bias (or ‘other’ bias) was assessed in terms of the appropriateness of statistical analyses, and the reporting of assumptions for statistical tests.

This review included a variety of study designs (for example, 19 of the 34 studies included clinical groups). Therefore, risk of bias was reported for study aspects relevant to this review. Subsequently, selection bias was assessed in terms of participant recruitment methods. Additionally, attrition/exclusion bias was assessed in terms of outcome data for the control group, or groups of interest for this study.

**Figure S1**

*Risk of Bias in Individual Studies*
