# Supplementary material for: The development of neural responses to emotional faces: A review of evidence from event-related potentials during early and middle childhood
Source: Dev Cogn Neurosci. 2021 Jul 21;51:100992. doi: 10.1016/j.dcn.2021.100992 (PMC8339225; doi:10.1016/j.dcn.2021.100992)
Supplement: Supplementary file 2 [file mmc2.docx]

**Table S1**

*Values Included for P100 Latency Meta-Regression Using the Median, Minimum, and Maximum Standard Errors*

| Dataset | Age | P100 Latency | Median | | Minimum | | Maximum | |
| --- | --- | --- | --- | --- | --- | --- | --- | --- |
|  |  |  | SE | IV weight | SE | IV weight | SE | IV weight |
| Apicella et al. (2013) | 9.70 | 104.23 ^b^ | 6.64 ^b^ | 0.004 | 6.64 | 0.004 | 6.64 | 0.006 |
| Batty and Taylor (2006) |  |  |  |  |  |  |  |  |
| Group 1 | 4.80 | 117.86 ^c^ | 1.98 ^c^ | 0.005 | 1.98 | 0.005 | 1.98 | 0.008 |
| Group 2 | 7.03 | 114.13 ^c^ | 2.10 ^c^ | 0.005 | 2.10 | 0.005 | 2.10 | 0.008 |
| Group 3 | 8.92 | 109.34 ^c^ | 2.09 ^c^ | 0.005 | 2.09 | 0.005 | 2.09 | 0.008 |
| Group 4 | 11.13 | 103.54 ^c^ | 2.02 ^c^ | 0.005 | 2.02 | 0.005 | 2.02 | 0.008 |
| Group 5 | 12.92 | 108.13 ^c^ | 1.96 ^c^ | 0.005 | 1.96 | 0.005 | 1.96 | 0.008 |
| Batty et al. (2011) |  |  |  |  |  |  |  |  |
| Group 1 | 10.51 | 103.00 ^b^ | 1.81 ^b^ | 0.005 | 1.81 | 0.005 | 1.81 | 0.008 |
| Group 2 | 7.70 | 108.71 ^c^ | 2.52 ^c^ | 0.005 | 2.52 | 0.005 | 2.52 | 0.008 |
| Curtis and Cicchetti (2011) | 3.53 | 134.45 ^c^ | 2.21^d^ | 0.005 | 1.81^d^ | 0.005 | 9.03^d^ | 0.005 |
| D'Hondt et al. (2017) | 4.58 | 141.67 ^b^ | 5.34 ^b^ | 0.004 | 5.34 | 0.004 | 5.34 | 0.007 |
| Dennis, et al (2009) | 6.82 | 135.22 ^b^ | 9.03 ^b^ | 0.004 | 9.03 | 0.003 | 9.03 | 0.005 |
| Hum, et al (2013a) | 10.14 | 142.09 ^c^ | 2.21^d^ | 0.005 | 1.81^d^ | 0.005 | 9.03^d^ | 0.005 |
| Hum, et al (2013b) | 10.13 | 138.91 ^c^ | 2.21^d^ | 0.005 | 1.81^d^ | 0.005 | 9.03^d^ | 0.005 |
| Luyster, et al (2017) | 12.29 | 99.03 ^a^ | 3.52 ^a^ | 0.005 | 3.52 | 0.004 | 3.52 | 0.007 |
| Meaux et al. (2014) |  |  |  |  |  |  |  |  |
| Group 1 | 5.08 | 111.71 ^a^ | 3.75 ^a^ | 0.005 | 3.75 | 0.004 | 3.75 | 0.007 |
| Group 2 | 6.93 | 105.04 ^a^ | 1.49 ^a^ | 0.005 | 1.49 | 0.005 | 1.49 | 0.008 |
| Group 3 | 8.98 | 109.09 ^a^ | 3.26 ^a^ | 0.005 | 3.26 | 0.004 | 3.26 | 0.007 |
| Miki et al. (2011) |  |  |  |  |  |  |  |  |
| Group 1 | 9.30 | 135.64 ^b^ | 2.00 ^b^ | 0.005 | 2.00 | 0.005 | 2.00 | 0.008 |
| Group 2 | 12.70 | 132.15 ^b^ | 2.21 ^b^ | 0.005 | 2.21 | 0.005 | 2.21 | 0.008 |
| Simonetti et al. (2019) | 12.15 | 114.85 ^b^ | 2.58 ^b^ | 0.005 | 2.58 | 0.005 | 2.58 | 0.008 |
| Tye et al. (2014) | 10.56 | 141.00 ^b^ | 2.21^d^ | 0.005 | 1.81^d^ | 0.005 | 9.03^d^ | 0.005 |
| Wong, et al. (2008) | 8.50 | 116.56 ^c^ | 2.21^d^ | 0.005 | 1.81^d^ | 0.005 | 9.03^d^ | 0.005 |
| Young, et al. (2017) | 12.70 | 120.68 ^b^ | 2.62 ^b^ | 0.005 | 2.62 | 0.005 | 2.62 | 0.008 |

Note. SE = standard error; Age = average age across dataset measured in years; P100 Latency = average P100 Latency across dataset measured in ms post stimulus onset

_a_ = value received from author; _b_ = value stated in article; _c_ = value estimated using plot digitizer; _d_ = value required imputation

**Table S2**

*Values Included for P100 Amplitude Meta-Regression Using the Median, Minimum, and Maximum Standard Error*

| Dataset | Age | P100 Amplitude | Median | | Minimum | | Maximum | |
| --- | --- | --- | --- | --- | --- | --- | --- | --- |
|  |  |  | SE | IV weight | SE | IV weight | SE | IV weight |
| Apicella et al. (2013) | 9.70 | 10.31^b^ | 1.78 ^b^ | 0.030 | 1.78 | 0.052 | 1.78 | 0.029 |
| Batty and Taylor (2006) |  |  |  |  |  |  |  |  |
| Group 1 | 4.80 | 23.41^c^ | 1.11 ^c^ | 0.032 | 1.11 | 0.058 | 1.11 | 0.031 |
| Group 2 | 7.03 | 24.44 ^c^ | 1.23 ^c^ | 0.032 | 1.23 | 0.057 | 1.23 | 0.030 |
| Group 3 | 8.92 | 19.61 ^c^ | 1.32 ^c^ | 0.032 | 1.32 | 0.057 | 1.32 | 0.030 |
| Group 4 | 11.13 | 16.14 ^c^ | 1.36 ^c^ | 0.032 | 1.36 | 0.056 | 1.36 | 0.030 |
| Group 5 | 12.92 | 14.74 ^c^ | 1.18 ^c^ | 0.032 | 1.18 | 0.058 | 1.18 | 0.030 |
| Batty et al. (2011) |  |  |  |  |  |  |  |  |
| Group 1 | 10.51 | 22.00 ^b^ | 1.94 ^b^ | 0.030 | 1.94 | 0.051 | 1.94 | 0.028 |
| Group 2 | 7.70 | 27.50 ^b^ | 2.97 ^b^ | 0.026 | 2.97 | 0.040 | 2.97 | 0.025 |
| Curtis and Cicchetti (2011) | 3.53 | 18.13 ^b^ | 1.08 ^b^ | 0.032 | 1.08 | 0.058 | 1.08 | 0.031 |
| D'Hondt et al. (2017) | 4.58 | 13.88 ^b^ | 1.66 ^b^ | 0.031 | 1.66 | 0.053 | 1.66 | 0.029 |
| Dennis, et al (2009) | 6.82 | 15.11 ^b^ | 1.52 ^b^ | 0.032 | 1.52 | 0.055 | 1.52 | 0.030 |
| Hum, et al (2013a) | 10.14 | 14.85 ^c^ | 1.19 ^c^ | 0.033 | 1.19 | 0.058 | 1.19 | 0.030 |
| Hum, et al (2013b) | 10.13 | 14.62 ^c^ | 0.29 ^b^ | 0.030 | 0.29 | 0.062 | 0.29 | 0.032 |
| Luyster, et al (2017) | 12.29 | 12.53 ^a^ | 1.84 ^a^ | 0.030 | 1.84 | 0.052 | 1.84 | 0.029 |
| Meaux et al. (2014) |  |  |  |  |  |  |  |  |
| Group 1 | 5.08 | 26.33 ^a^ | 3.22 ^a^ | 0.025 | 3.22 | 0.038 | 3.22 | 0.024 |
| Group 2 | 6.93 | 20.30 ^a^ | 2.58 ^a^ | 0.027 | 2.58 | 0.044 | 2.58 | 0.026 |
| Group 3 | 8.98 | 15.20 ^a^ | 2.13 ^a^ | 0.029 | 2.13 | 0.049 | 2.13 | 0.028 |
| Miki et al. (2011) |  |  |  |  |  |  |  |  |
| Group 1 | 9.30 | 6.68 ^b^ | 0.70 ^b^ | 0.033 | 0.70 | 0.061 | 0.70 | 0.031 |
| Group 2 | 12.70 | 3.12 ^b^ | 0.74 ^b^ | 0.033 | 0.74 | 0.061 | 0.74 | 0.031 |
| Simonetti et al. (2019) | 12.15 | 9.27 ^c^ | 1.36^d^ | 0.032 | 0.29^d^ | 0.062 | 2.97^d^ | 0.025 |
| Tye et al. (2014) | 10.56 | 9.69 ^c^ | 1.36^d^ | 0.032 | 0.29^d^ | 0.062 | 2.97^d^ | 0.025 |
| Wong, et al. (2008) | 8.50 | 11.02 ^c^ | 1.36^d^ | 0.032 | 0.29^d^ | 0.062 | 2.97^d^ | 0.025 |
| Young, et al. (2017) | 12.70 | 10.05 ^b^ | 0.81 ^b^ | 0.033 | 0.81 | 0.060 | 0.81 | 0.031 |

Note. SE = standard error; Age = average age across dataset measured in years; P100 Amplitude = average P100 amplitude across dataset measured in µV

_a_ = value received from author; _b_ = value stated in article; _c_ = value estimated using plot digitizer; _d_ = value required imputation

**Table S3**

*Values Included for N170 Latency Meta-Regression Using the Median Minimum, and Maximum Standard Error*

| Dataset | Age | N170 Latency | Median | | Minimum | | Maximum | |
| --- | --- | --- | --- | --- | --- | --- | --- | --- |
|  |  |  | SE | IV weight | SE | IV weight | SE | IV weight |
| Apicella et al. (2013) | 9.70 | 168.13^b^ | 6.96 ^b^ | 0.00049 | 6.96 | 0.00049 | 6.96 | 0.00048 |
| Battaglia et al. (2007) | 8.85 | 152.94 ^b^ | 1.17 ^b^ | 0.0005 | 1.17 | 0.0005 | 1.17 | 0.00049 |
| Battaglia et al. (2017) | 9.23 | 115.18 ^a^ | 0.91 ^a^ | 0.0005 | 0.91 | 0.0005 | 0.91 | 0.00049 |
| Batty and Taylor (2006) |  |  |  |  |  |  |  |  |
| Group 1 | 4.80 | 216.75 ^c^ | 4.64 ^c^ | 0.00049 | 4.64 | 0.0005 | 4.64 | 0.00049 |
| Group 2 | 7.03 | 212.58 ^c^ | 5.33 ^c^ | 0.00049 | 5.33 | 0.0005 | 5.33 | 0.00049 |
| Group 3 | 8.92 | 189.39 ^c^ | 4.41 ^c^ | 0.00049 | 4.41 | 0.0005 | 4.41 | 0.00049 |
| Group 4 | 11.13 | 151.37 ^c^ | 5.80 ^c^ | 0.00049 | 5.80 | 0.00049 | 5.80 | 0.00049 |
| Group 5 | 12.92 | 152.76 ^c^ | 4.17 ^c^ | 0.00049 | 4.17 | 0.0005 | 4.17 | 0.00049 |
| Batty et al. (2011) |  |  |  |  |  |  |  |  |
| Group 1 | 10.51 | 175.00 ^b^ | 9.04 ^b^ | 0.00048 | 9.04 | 0.00048 | 9.04 | 0.00048 |
| Group 2 | 7.70 | 195.64 ^c^ | 8.64 ^c^ | 0.00048 | 8.64 | 0.00048 | 8.64 | 0.00048 |
| Curtis and Cicchetti (2011) | 3.53 | 213.50 ^c^ | 5.33 ^d^ | 0.00049 | 4.17^d^ | 0.0005 | 9.04^d^ | 0.00048 |
| D'Hondt et al. (2017) | 4.58 | 219.00 ^b^ | 5.08 ^b^ | 0.00049 | 5.08 | 0.0005 | 5.08 | 0.00049 |
| Dennis, et al (2009) | 6.82 | 188.01 ^b^ | 5.33^d^ | 0.00049 | 4.17^d^ | 0.0005 | 9.04^d^ | 0.00048 |
| Hoyniak et al. (2019) | 4.13 | 219.76 ^a^ | 4.38 ^a^ | 0.00049 | 4.38 | 0.0005 | 4.38 | 0.00049 |
| Luyster, et al (2017) | 12.29 | 156.60 ^a^ | 5.73 ^a^ | 0.00049 | 5.73 | 0.00049 | 5.73 | 0.00049 |
| Magnuson, et al (2020) | 9.60 | 274.53 ^a^ | 4.87 ^a^ | 0.00049 | 4.87 | 0.0005 | 4.87 | 0.00049 |
| Meaux et al. (2014) |  |  |  |  |  |  |  |  |
| Group 1 | 5.08 | 227.30 ^a^ | 8.68 ^a^ | 0.00048 | 8.68 | 0.00048 | 8.68 | 0.00048 |
| Group 2 | 6.93 | 200.66 ^a^ | 2.83 ^a^ | 0.0005 | 2.83 | 0.0005 | 2.83 | 0.00049 |
| Group 3 | 8.98 | 205.63 ^a^ | 4.76 ^a^ | 0.00049 | 4.76 | 0.0005 | 4.76 | 0.00049 |
| Miki et al. (2011) |  |  |  |  |  |  |  |  |
| Group 1 | 9.30 | 236.07 ^b^ | 5.96 ^b^ | 0.00049 | 5.96 | 0.00049 | 5.96 | 0.00049 |
| Group 2 | 12.70 | 222.93 ^b^ | 7.55 ^b^ | 0.00048 | 7.55 | 0.00049 | 7.55 | 0.00048 |
| O'Toole, et al. (2013) | 6.19 | 211.61 ^a^ | 2.49^a^ | 0.0005 | 2.49 | 0.0005 | 2.49 | 0.00049 |
| Tye et al. (2014) | 10.56 | 207.00 ^b^ | 5.33^d^ | 0.00049 | 4.17^d^ | 0.0005 | 9.04^d^ | 0.00048 |
| Wong, et al. (2008) | 8.50 | 184.62 ^c^ | 5.33^d^ | 0.00049 | 4.17^d^ | 0.0005 | 9.04^d^ | 0.00048 |
| Young, et al. (2017) | 12.70 | 165.37 ^c^ | 2.71^b^ | 0.0005 | 2.71 | 0.0005 | 2.71 | 0.00049 |

Note. SE = standard error; Age = average age across dataset measured in years; N170 Latency = average N170 Latency across dataset measured in ms post stimulus onset

_a_ = value received from author; _b_ = value stated in article; _c_ = value estimated using plot digitizer; _d_ = value required imputation

**Table S4**

*Values Included for N170 Amplitude Meta-Regression Using the Median Minimum, and Maximum Standard Error*

| Dataset | Age | N170 Amplitude | Median | | Minimum | | Maximum | |
| --- | --- | --- | --- | --- | --- | --- | --- | --- |
|  |  |  | SE | IV weight | SE | IV weight | SE | IV weight |
| Apicella et al. (2013) | 9.70 | -0.08 ^b^ | 1.22 ^b^ | 0.040 | 1.22 | 0.042 | 1.22 | 0.038 |
| Battaglia et al. (2007) | 8.85 | -11.29 ^b^ | 0.52 ^b^ | 0.042 | 0.52 | 0.044 | 0.52 | 0.040 |
| Battaglia et al. (2017) | 9.23 | -11.31 ^a^ | 0.38 ^a^ | 0.043 | 0.38 | 0.044 | 0.38 | 0.040 |
| Batty and Taylor (2006) |  |  |  |  |  |  |  |  |
| Group 1 | 4.80 | -4.81 ^c^ | 0.91^c^ | 0.041 | 0.91 | 0.043 | 0.91 | 0.039 |
| Group 2 | 7.03 | -6.77 ^c^ | 0.82 ^c^ | 0.041 | 0.82 | 0.043 | 0.82 | 0.039 |
| Group 3 | 8.92 | -5.80 ^c^ | 0.87 ^c^ | 0.041 | 0.87 | 0.043 | 0.87 | 0.039 |
| Group 4 | 11.13 | -4.09 ^c^ | 0.94 ^c^ | 0.041 | 0.94 | 0.043 | 0.94 | 0.039 |
| Group 5 | 12.92 | -2.63 ^c^ | 0.82 ^c^ | 0.041 | 0.82 | 0.043 | 0.82 | 0.039 |
| Batty et al. (2011) |  |  |  |  |  |  |  |  |
| Group 1 | 10.51 | -10.25 ^c^ | 0.93^d^ | 0.041 | 0.72^d^ | 0.043 | 1.61^d^ | 0.037 |
| Group 2 | 7.70 | -7.73 ^c^ | 0.93^d^ | 0.041 | 0.72^d^ | 0.043 | 1.61^d^ | 0.037 |
| Bertoletti, et al. (2012) |  |  |  |  |  |  |  |  |
| Group 1 | 8.74 | -8.04 ^b^ | 0.53 ^b^ | 0.042 | 0.53 | 0.044 | 0.53 | 0.040 |
| Group 2 | 9.08 | -10.64 ^b^ | 0.72 ^b^ | 0.042 | 0.72 | 0.043 | 0.72 | 0.040 |
| Curtis and Cicchetti (2011) | 3.53 | 9.10 ^b^ | 1.46 ^b^ | 0.039 | 1.46 | 0.041 | 1.46 | 0.037 |
| D'Hondt et al. (2017) | 4.58 | -0.30 ^b^ | 1.61 ^b^ | 0.038 | 1.61 | 0.040 | 1.61 | 0.037 |
| Dennis, et al (2009) | 6.82 | 11.48 ^b^ | 1.46 ^b^ | 0.039 | 1.46 | 0.041 | 1.46 | 0.037 |
| Hoyniak et al. (2019) | 4.13 | 3.29 ^a^ | 1.01 ^a^ | 0.041 | 1.01 | 0.042 | 1.01 | 0.039 |
| Hum, et al (2013a) | 10.14 | -1.18 ^c^ | 0.63 ^b^ | 0.042 | 0.63 | 0.044 | 0.63 | 0.040 |
| Luyster, et al (2017) | 12.29 | -1.22 ^a^ | 1.32 ^a^ | 0.040 | 1.32 | 0.041 | 1.32 | 0.038 |
| Magnuson, et al (2020) | 9.60 | 1.08 ^a^ | 0.63 ^a^ | 0.042 | 0.63 | 0.044 | 0.63 | 0.040 |
| Meaux et al. (2014) |  |  |  |  |  |  |  |  |
| Group 1 | 5.08 | -10.11 ^a^ | 2.07 ^a^ | 0.036 | 2.07 | 0.037 | 2.07 | 0.034 |
| Group 2 | 6.93 | -3.85 ^a^ | 1.01 ^a^ | 0.041 | 1.01 | 0.042 | 1.01 | 0.039 |
| Group 3 | 8.98 | -4.84 ^a^ | 0.87 ^a^ | 0.041 | 0.87 | 0.043 | 0.87 | 0.039 |
| Miki et al. (2011) |  |  |  |  |  |  |  |  |
| Group 1 | 9.30 | -8.16 ^b^ | 0.53 ^b^ | 0.042 | 0.53 | 0.044 | 0.53 | 0.040 |
| Group 2 | 12.70 | -7.25 ^b^ | 0.50 ^b^ | 0.042 | 0.50 | 0.044 | 0.50 | 0.040 |
| O'Toole, et al. (2013) | 6.19 | -0.39 ^a^ | 0.97 ^a^ | 0.041 | 0.97 | 0.043 | 0.97 | 0.039 |
| Tye et al. (2014) | 10.56 | -6.28 ^c^ | 0.93^d^ | 0.041 | 0.72^d^ | 0.043 | 1.61^d^ | 0.037 |
| Wong, et al. (2008) | 8.50 | -3.66 ^c^ | 0.93^d^ | 0.041 | 0.72^d^ | 0.043 | 1.61^d^ | 0.037 |
| Young, et al. (2017) | 12.70 | -0.55 ^b^ | 0.54 ^b^ | 0.042 | 0.54 | 0.044 | 0.54 | 0.040 |

Note. SE = standard error; Age = average age across dataset measured in years; N170 Amplitude = average N170 amplitude across dataset measured in µV

_a_ = value received from author; _b_ = value stated in article; _c_ = value estimated using plot digitizer; _d_ = value required imputation
